# Fish oil users of Greece: Predictors, knowledge and habits regarding dietary supplement use

**DOI:** 10.3934/publichealth.2023058

**Published:** 2023-11-13

**Authors:** Panagiotis-David Soukiasian, Zacharenia Kyrana, Konstantina Gerothanasi, Efstratios Kiranas, Lambros E. Kokokiris

**Affiliations:** 1 Department of Nutritional Sciences and Dietetics, School of Health Sciences, International Hellenic University, 57400 Thessaloniki, Greece; 2 Laboratory of Agronomy, Faculty of Agriculture, Forestry and Natural Environment, School of Agriculture, Aristotle University of Thessaloniki, 54124 Thessaloniki, Greece

**Keywords:** dietary supplements use, fish oil supplements, consumer stance, perception population, Greece

## Abstract

**Background:**

Fish oil (FO) dietary supplements (DS) have gained popularity over the past few decades and emerged as one of the most popular DS in both Europe and the USA. However, in Greece, no study has been carried out to determine the prevalence and characteristics of FO consumers.

**Objective:**

The aim of this study was to describe the stance (i.e., beliefs, knowledge and habits) and practices of FO users in Greece towards DS.

**Methods:**

A cross-sectional study, using in-person questionnaires and a stratified random sampling technique, was conducted throughout 2018–2019. A total of 28491 Greek citizens over 15 years old were interviewed in 74 regional units corresponding to the 13 administrative regions of Greece. Having used DS at least once, deemed one as: DS+FO, if FO were among them; DS-FO, if FO were never used or DS non-user, if DS were never used. Pearson's chi-square test was used to determine independence between relevant outcome variables and FO use and multinomial logistic regression was performed to create models predictive of FO use.

**Results:**

The sample consisted of 3.5% DS+FO, 51.3% DS-FO and 43.8% DS non-users, while 1.4% could not remember whether they had used DS. Significant predictors for being a DS+FO were identified. DS+FO were more likely to judge a DS-less diet as insufficient, support DS use and comprehend DS's labels. Also, DS+FO had used additional DS, considered more parameters when buying DS and were receivers of DS recommendations from more sources compared to DS-FO. 90% of DS users seek at least one approval source of their DS and 50% support DS recommendation by doctors.

**Conclusion:**

Favorableness towards DS is more prevalent among DS+FO. Most respondents lack knowledge about the legislative background of DS and many would agree to professional guidance.

## 1. Introduction

Dietary supplements (DS) have earned a place in the basket of consumers during the last decades [1]–[10]; fish oils (FO) have not been an exception [2]–[6],[8],[10]. Generally, multivitamins seem to dominate [1]–[3],[6],[7],[9],[11]. However, even if FO are not as not as widely used in Greece [9],[12], they have been shown to be, if not the most consumed DS [4],[5],[10], one of the most consumed DS [2],[3],[6],[11], especially in countries like England [6],[8],[10] and Norway [8]. In fact, in the US, the use of FO saw an increase from 1.3% to 12.0% during 1999–2012 [13], and they have been characterized to be among the most used non-vitamin, non-mineral DS [14].

FO have been around for centuries. Hippocrates (400 BC) mentioned their medicinal use [15], Vikings (700–1000AD) consumed them during wintertime when sunlight was not sufficient [16], and fishermen of northern coastal Europe consumed them for many years [15]. Indeed, Norwegians are known for traditional consumption of FO [8], with the latest Nordic Nutrition Recommendations (2012) recommending their consumption, considering FO not as DS but as food [17].

FO are essentially considered nutraceuticals and contain essential omega-3 polyunsaturated fatty acids (*ω*-3), like eicosapentaenoic (EPA) and docosahexaenoic acid (DHA), although vitamins might be added (e.g., vitamin *E*) or be included naturally e.g., vitamin *A* and *D* in cod liver oil [18]. Additionally, they seem to possess anti-inflammatory properties [19], beneficial to human health, such as their contribution towards endothelial function [20]. In fact, health claims such as the maintenance of normal blood pressure, heart function and triglyceride levels have been authorized by the European Health Authority (EFSA) to be used by DS manufacturers [21].

Given the popularity of DS, differences between DS users and non-users have been explored. Specifically, predictors of use, behaviors, attitudes and general stances have been examined. For example, being a woman [2],[9],[22]–[25], having a higher level of education [2],[9],[23],[25] and following certain dietary patterns [9],[24],[25] have been shown to predict DS use. Additionally, DS users seem to be more likely to hold positive views towards DS, compared to DS non-users [9],[26]. Differences between FO users and DS non-users have been examined in many countries such as New Zealand [3], Australia [27] and the UK [10]. Indeed, significant behavioral and other differences (e.g., sources of DS recommendation) and influences of factors were detected. However, no such study has yet been carried out in Greece. Therefore, we aimed to explore the aforementioned topics. Our research questions evolved around whether there are any differences between DS users who had used FO in the past (e.g., DS+FO), DS users who had never used FO (DS-FO) and DS non-users with regards to demographic characteristics, DS label comprehension and opinions about DS use and diet. Furthermore, we aimed to look into whether DS+FO differed with regards to the quantity and type of DS they consumed, sources of information or recommendations about DS, sources they consulted for DS approval and parameters they considered when buying DS.

## 2. Material and methods

### 2.1. Study design

The current investigation was based on questionnaires that were distributed from 2018 to 2019 as part of the project titled “Creation of a database in the Department of Nutrition and Dietetics to investigate the nutrition habits of Greek consumers and their relationship with nutrition supplements and the nutrition label”, conducted at the Department of Nutrition and Dietetics of the International Hellenic University. The in-person surveys were carried out by the NUTSTUDY team, consisting of 90 trained senior students and their professors from the Department of Nutritional Sciences and Dietetics at the International Hellenic University (IHU). The research protocol was approved by the Committee for Research Ethics (IHU).

The questionnaire consisted of closed-ended questions, divided into different sections. The first was about the demographic characteristics of respondents [i.e. sex, age, BMI (calculated according to the declared weight and height), income, educational level, employment status, exercise (exercisers: exercise was reportedly performed at least once per week; and, non-exercisers: exercise was reported as “rarely” or “never”), type of diet], the second was about the comprehension of DS labels and views about DS and diet, and the third was about the sources of DS approval, information and recommendation, the types of DS used and the DS purchasing parameters.

The verbal consent of participants was obtained following the provision of a research information sheet and a detailed explanation of the project's objectives. Subsequently, participants were instructed on the completion of the questionnaire. The target group was the Greek population and the sample collection method was proportional stratified random sampling. The Greek population was categorized into 74 regional units (strata), which align with the 13 administrative regions of Greece. The students visited various places within each regional unit (i.e., food stores, supermarkets, gyms and pharmacies) and they distributed the questionnaire to individuals aged at least 15 years old, randomly, without considering socio-economic status, educational level or any other inclusion criteria.

The collected sample was representative of the general population in terms of sex. Specifically, according to the 2011 census, the Greek population consisted of 51% women and 49% men. Accordingly, our sample consisted of 53% women and 47% men. The initial sample consisted of 31824 Greek citizens. The questionnaires that were incomplete were removed, leaving 28491 respondents. Those that had used a DS at least once in their lifetime were defined as DS users (15608; 54.8%) and those that had never used DS as DS non-users (12494; 43.8%). The remaining 389 (1.4%) respondents could not remember whether they had used a DS or not and were not considered when analyzing the outcome variables, leading to a final sample size of 28102. Among DS users, if FO were among their reported DS, they were defined as DS+FO (1001; 6.5%); otherwise, they were defined as DS-FO (14607; 93.5%).

The assumed definition of DS was as follows: “*foodstuffs the purpose of which is to supplement the normal diet and which are concentrated sources of nutrients or other substances with a nutritional or physio-logical effect, alone or in combination, marketed in dose form, namely forms such as capsules, pastilles, tablets, pills and other similar forms, sachets of powder, ampoules of liquids, drop dispensing bottles, and other similar forms of liquids and powders de-signed to be taken in measured small unit quantities*” (Directive 2002/46/EC, European Parliament and Council, 2002) [28].

### 2.2. Statistical analysis

For all of the variables, the frequencies and percentages were presented overall and according to sex, age, body mass index (BMI), monthly income, education level, employment status, exercise, type of adhered diet and DS use. Pearson's chi-square (χ^2^) test was used to detect for independence or significant association between subgroups of the categorical variables [29] and column proportions were compared using z test.

Additionally, multinomial logistic regression (MLR) was performed [30], while all of the analysis' conditions were examined, in order to create a general predictive profile of a DS+FO. The dependent variable was the status of DS use, having three mutually exclusive and exhaustive categories (“DS non-users”, “DS-FO” and “DS+FO”). The reference category of the dependent variable was the DS+FO. The independent variables were the nominal variables of the demographic characteristics of respondents (i.e., sex, age, BMI, etc.). All of the assumptions of the MLR were examined and were valid. Additionally, in the MLR analysis, there were two types of models. The first model was the null one because it includes only the intercept variable. The second model was the final (or the full) one, which includes all the independent variables. The Akaike and the Bayesian information criteria (AIC and BIC) showed that the final model had better fit than the null (AIC_null_ = 20114.9 > AIC_final_ = 18801.2 and BIC_null_ = 20131.4 > BIC_final_ = 19295.8). Also, the fit of the final model was significant (χ^2^ of Pearson = 1429.7, *p* < 0.001) and correctly predicted 57.4% of the cases. Furthermore, all the independent variables were significant for the final model (χ^2^ test of Pearson, *p* < 0.05).

The original SPSS output of the MLR analysis with regards to the calculation of the OR of one being a DS non-user or a DS-FO compared to being a DS+FO (reference category) can be found on Supplementary Table S7. However, we assumed that showing the odds of the opposite, i.e., being a DS+FO compared to being a DS non-user or DS-FO, would lead to more comprehensive results (transformed results). Therefore, Supplementary Table S7 was modified, i.e., all the originally generated OR values were modified using Formula 1.

$$OR_{modified}=\frac{1}{OR_{original}}$$ (1)

Afterwards, the modified OR values were expressed as percentages using Formula 2.

$$OR_{modified\;as\;\%}=\left[OR_{modified}-1\right]\times 100$$ (2)

Thus, percentages with a negative sign indicate fewer odds of being a DS+FO compared to being a DS non-user or a DS-FO, while those with a positive sign indicate higher odds. Henceforth, the following references to OR refer exclusively on their calculated form (Table 6) and not in the original form presented in Supplementary Table S7.

All analyses were performed using IBM SPSS Statistics version 29.0. In order to mitigate the risk of a type I error, which entails an increased probability of obtaining a significant result purely by chance, a Bonferroni correction was applied to adjust *p*-values in all multiple analyses performed. The significance level was set at *α* = 0.05.

## 3. Results

The analyzed sample consisted of 28102 respondents, consisting of 12494 (44.5%) DS non-users, 1001 (3.6%) DS+FO and 14607 (52.0%) DS-FO.

### 3.1. Demographic characteristics

Overall, our sample consisted of slightly more women (53.0% *vs*. 47.0%), 21–50 years olds (72.9%), those with normal BMI (54.5%), monthly income up to 1000 € (81.0%), secondary education (41.4%), tertiary education (45.0%), students (25.8%), private employees (25.2%), public employees (13.7%), freelancers (16.8%), farmers (4.7%), unemployed individuals (13.8%), exercisers (56.7%) and followers of a mixed unrestricted diet (animal and plant foods) (67.0%).

Regarding DS+FO users, 50.6% were men and consisted mainly by those between 21–40 years old (58.4%), respondents with normal BMI (51.3%) or overweight (34.8%), monthly income up to 1000 € (73.3%), secondary education (35.7%), tertiary education (48.2%), private employees (27.8%), public employees (17.9%), students (20.4%), freelancers (19.4%), farmers (2.8%), unemployed individuals (11.8%), exercisers (67.6%) and followers of mixed unrestricted diets (60.4%).

There was a significant association of the demographic characteristics and the DS use categories (i.e., DS+FO, DS-FO and DS non-users, *p* = 0.000, Table 1). Significantly different percentages of DS+FO compared to DS-FO were observed in some variables which were found to be strongly associated with the DS use category. For example, significantly different percentages were found in sex (among DS+FO: higher percentage of men, i.e. 50.6% *vs*. 43.8%, Table 1), age (among DS+FO: higher percentages of older respondents), BMI (among DS+FO: slightly higher percentages of underweight and overweight respondents, but also lower percentages of respondents with normal weight), income (among DS+FO: higher percentages of respondents with high income), education (among DS+FO: slightly higher prevalence of postgraduate education but a lower one regarding secondary education) and exercise (among DS+FO: higher prevalence of exercisers, i.e. 67.6% *vs*. 61.2%). However, DS+FO and DS-FO, with regards to their employment status or type of adhered diet, were similar or, at most, slightly different from each other.

**Table 1.** Absolute and relative frequencies in parenthesis (%) of the demographic characteristics of respondents, based on total respondents (Total), dietary supplement (DS) users who had used fish oils (FO) among other DS (DS+FO), DS users who had used DS but not FO (DS-FO) and DS non-users who had never used DS. The *p*-values of chi-square tests of independence between the demographic characteristics of respondents, and the DS use categories (i.e., DS+FO, DS-FO and DS non-users) after adjustment with the Bonferroni correction test are presented. Within a row, column proportions that do not share any common superscript letters are significantly different at the α = 0.05 level (column proportions compared with the z test; p value adjusted with the Bonferroni method).

**Demographic characteristics**	**Total ** **(n = 28102)**	**DS+FO ** **(n = 1001)**	**DS-FO ** **(n = 14607)**	**DS non-users ** **(n = 12494)**	***p*-value**
**Sex**					0.000
Man	13199 (47.0)	507 (50.6)^a^	6400 (43.8)^b^	6292 (50.4)^a^	
Woman	14903 (53.0)	494 (49.4)^a^	8207 (56.2)^b^	6202 (49.6)^a^	
**Age** (years old)					0.000
15–20	3861 (13.7)	80 (8.0)^a^	1701 (11.6)^b^	2080 (16.6)^c^	
21–30	10457 (37.2)	341 (34.1)^a^	5744 (39.3)^b^	4372 (35.0)^a^	
31–40	5835 (20.8)	243 (24.3)^a^	3315 (22.7)^a^	2277 (18.2)^b^	
41–50	4191 (14.9)	168 (16.8)^a^	2185 (15.0)^a^	1838 (14.7)^a^	
51–60	2597 (9.2)	125 (12.5)^a^	1201 (8.2)^b^	1271 (10.2)^c^	
>60	1161 (4.1)	44 (4.4)^a^	461 (3.2)^b^	656 (5.3)^a^	
**BMI**					0.000
Underweight	837 (3.0)	44 (4.4)^a^	462 (3.2)^b^	331 (2.6)^c^	
Normal weight	15319 (54.5)	514 (51.3)^a^	8106 (55.5)^b^	6699 (53.6)^a^	
Overweight	9132 (32.5)	348 (34.8)^a^	4618 (31.6)^b^	4166 (33.3)^a^	
Obese	2814 (10.0)	95 (9.5)^a^	1421 (9.7)^a^	1298 (10.4)^a^	
**Monthly income (€)**					0.000
<500	12382 (44.1)	369 (36.9)^a^	6042 (41.4)^b^	5971 (47.8)^c^	
500–1000	10357 (36.9)	364 (36.4)^a,b^	5707 (39.1)^b^	4286 (34.3)^a^	
1001–1500	4008 (14.3)	185 (18.5)^a^	2140 (14.7)^b^	1683 (13.5)^c^	
1501–2000	799 (2.8)	47 (4.7)^a^	415 (2.8)^b^	337 (2.7)^b^	
>2000	556 (2.0)	36 (3.6)^a^	303 (2.1)^b^	217 (1.7)^c^	
**Education level**					0.000
Primary	1340 (4.8)	34 (3.4)^a^	449 (3.1)^a^	857 (6.9)^b^	
Secondary	11629 (41.4)	357 (35.7)^a^	5853 (40.1)^b^	5419 (43.4)^c^	
Tertiary	12659 (45.0)	482 (48.2)^a^	6855 (46.9)^a^	5322 (42.6)^b^	
Postgraduate	2474 (8.8)	128 (12.8)^a^	1450 (9.9)^b^	896 (7.2)^c^	
**Employment status**					0.008
Unemployed	3880 (13.8)	118 (11.8)^a^	1692 (11.6)^a^	2070 (16.6)^b^	
Student	7254 (25.8)	204 (20.4)^a^	3713 (25.4)^b^	3337 (26.7)^c^	
Private employee	7094 (25.2)	278 (27.8)^a^	4014 (27.5)^a^	2802 (22.4)^b^	
Public employee	3838 (13.7)	179 (17.9)^a^	2034 (13.9)^b^	1625 (13.0)^c^	
Freelancer	4727 (16.8)	194 (19.4)^a^	2619 (17.9)^a^	1914 (15.3)^b^	
Farmer	1309 (4.7)	28 (2.8)^a^	535 (3.7)^a^	746 (6.0)^b^	
**Exercise**					0.000
Exerciser	15925 (56.7)	677 (67.6)^a^	8943 (61.2)^b^	6305 (50.5)^c^	
Non-exerciser	12177 (43.3)	324 (32.4)^a^	5664 (38.8)^b^	6189 (49.5)^c^	
**Type of diet**					0.000
Mixed unrestricted	18836 (67.0)	605 (60.4)^a^	9269 (63.5)^a^	8962 (71.7)^b^	
Fat restricted	4293 (15.3)	171 (17.1)^a^	2505 (17.1)^a^	1617 (12.9)^b^	
Calorie restricted	2696 (9.6)	96 (9.6)^a,b^	1466 (10.0)^b^	1134 (9.1)^a^	
Starch/carbohydrate restricted	1090 (3.9)	68 (6.8)^a^	662 (4.5)^b^	360 (2.9)^c^	
Lacto-ovo-vegetarianism	504 (1.8)	24 (2.4)^a^	293 (2.0)^a^	187 (1.5)^b^	
Vegan/vegetarian	443 (1.6)	27 (2.7)^a^	269 (1.8)^a^	147 (1.2)^b^	
Lacto-vegetarianism	199 (0.7)	6 (0.6)^a,b^	118 (0.8)^b^	75 (0.6)^a^	
Other diet	41 (0.1)	4 (0.4)^a^	25 (0.2)^b^	12 (0.1)^b^	

Significantly different percentages of DS non-users compared to DS users overall (i.e. DS+FO, DS-FO) were found regarding age (among DS non-users: slightly higher percentages of young but lower percentages of old age respondents, Table 1), income (among DS non-users: significantly higher percentage of the <500 € group, Table 1), education (among DS non-users: higher percentages of primary and secondary educated respondents but lower of tertiary and postgraduate educated ones), employment (among DS non-users: higher percentages of the unemployed, student and farmer groups but lower of private/public employees and freelancers), exercise (lower percentage of exercised respondents, i.e. 50.5% in the DS non-users group *vs*. 67.6% & 61.2% for the DS+FO and DS-FO groups respectively, Table 1) and diet (among DS non-users: higher percentage, i.e., 71.7%, of mixed unrestricted diet but lower in the case of specific/restricted diets, i.e. fat, starch/carbohydrate, vegan, etc.).

### 3.2. DS label comprehension and views about DS and diet

The comparison between the three DS-use groups (DS+FO *vs*. DS-FO *vs*. DS non-users), regarding views about DS and DS label comprehension revealed certain differences (Table 2). Specifically, DS non-users were the most likely to firmly believe that nutrients from foods are enough to ensure good health, while DS+FO displayed the lowest level of agreement (25.6% *vs*. 27.9% *vs*. 44.5%, respectively). Conversely, DS non-users were approximately twice as likely to not know, by reading DS's labels, whether DS are of personal importance (15.0% *vs*. 15.7% *vs*. 31.4%) and whether DS or their ingredients are approved (34.0% *vs*. 37.1% *vs*. 72.7%), while DS+FO had the highest percentage of respondents who confidently reported comprehension for both of these matters (55.2% *vs*. 45.8% *vs*. 29.1% and 33.7% *vs*. 27.6% *vs*. 18.0%, respectively).

Additionally, DS non-users were the least likely to agree with DS-friendly statements, while DS+FO were the most likely to do so. Agreement with the idea of the recommendation of DS by doctors was most prevalent among DS non-users and the least prevalent among DS+FO (48.1% *vs*. 52.3% *vs*. 71.1%; Supplementary Table S1 displays the above data but with the frequencies of DS+FO and DS-FO pooled together).

**Table 2.** Absolute and relative frequencies in parenthesis (%) of DS label comprehension and views about DS, based on total respondents (Total), dietary supplement (DS) users who had used fish oils (FO) among other DS (DS+FO), DS users who had used DS but not FO (DS-FO) and DS non-users who had never used DS. The *p*-values of chi-square tests of independence between given answers and DS use categories (i.e., DS+FO, DS-FO and DS non-users) after adjustment with the Bonferroni correction test are presented. Within a row, column proportions that do not share any common superscript letters are significantly different at the α = 0.05 level (column proportions compared with the z test; p value adjusted with the Bonferroni method).

**DS label comprehension and views about DS**	**Total ** **(*n* = 28102)**	**DS+FO ** **(*n* = 1001)**	**DS-FO ** **(*n* = 14607)**	**DS non-users ** **(*n* = 12494)**	***p*-value**
**Are nutrients from foods enough to ensure good health?**					0.000
Yes	9891 (35.2)	256 (25.6)^a^	4081 (27.9)^a^	5554 (44.5)^b^	
Maybe yes	9835 (35.0)	309 (30.9)^a^	4930 (33.8)^a,b^	4596 (36.8)^b^	
No	6042 (21.5)	366 (36.6)^a^	4488 (30.7)^b^	1188 (9.5)^c^	
I don't know	2334 (8.3)	70 (7.0)^a,b^	1108 (7.6)^b^	1156 (9.3)^a^	
**Can you understand whether DS are important for you by reading their labels?**					0.000
Yes	10878 (38.7)	553 (55.2)^a^	6693 (45.8)^b^	3632 (29.1)^c^	
Maybe yes	10857 (38.6)	298 (29.8)^a^	5622 (38.5)^b^	4937 (39.5)^b^	
No	6367 (22.7)	150 (15.0)^a^	2292 (15.7)^a^	3925 (31.4)^b^	
**Can you recognize which ingredients or DS are approved if you read the DS's label?**					0.000
Yes	6614 (23.5)	337 (33.7)^a^	4029 (27.6)^b^	2248 (18.0)^c^	
Maybe yes	9142 (32.5)	323 (32.3)^a,b^	5163 (35.3)^b^	3656 (29.3)^a^	
No	12346 (43.9)	341 (34.0)^a^	5415 (37.1)^a^	6590 (72.7)^b^	
**With which of the following statements do you agree?^†^**				
DS are necessary for all ages	4061 (14.5)	269 (26.9)^a^	2743 (18.8)^b^	1049 (8.4)^c^	0.000
DS are generally harmless	7012 (25.0)	351 (35.1)^a^	4249 (29.1)^b^	2412 (19.3)^c^	0.000
Regular DS use can prevent many ailments	6083 (21.6)	328 (32.8)^a^	3677 (25.2)^b^	2078 (16.6)^c^	0.000
DS can prevent cancer	1276 (4.5)	79 (7.9)^a^	713 (4.9)^b^	484 (3.9)^c^	0.000
DS must be recommended by doctors	17007 (60.5)	481 (48.1)^a^	7640 (52.3)^b^	8886 (71.1)^c^	0.000
None of the above	275 (1.0)	5 (0.5)^a^	83 (0.6)^a^	187 (1.5)^b^	0.000

Note: ^†^Respondents could select more than one of the available statements.

### 3.3. Sources of approval, information and recommendation regarding DS

The top sought approval sources of DS were the National Organization for Medicines (NOM; 58.0%) and the supplier or pharmacist (27.6%), while 9.8% did not pay any attention to this matter (Table 3). Additionally, the top sources of information and recommendations were doctors (46.7% and 42.4%, respectively) and pharmacists (42.1% and 29.3%, respectively). The comparison between DS+FO and DS-FO shows that more DS+FO check whether DS are approved by NOM (64.7% *vs*. 57.6%) but fewer seek approval from the supplier or pharmacist (21.3% *vs*. 28.0%). However, 90.0% of DS+FO and DS-FO seek approval from at least one of the three listed sources (Supplementary Table S2).

**Table 3.** Absolute and relative frequencies in parenthesis (%) of DS sources of approval, information and recommendation based on total DS users (Total), dietary supplement (DS) users who had used fish oils (FO) among other DS (DS+FO) and DS users who had used DS but not FO (DS-FO). The *p*-values of chi-square tests of independence between the above sources and DS user categories (i.e., DS+FO, DS-FO) after adjustment with Bonferroni correction test, are presented. Within a row, column proportions that do not share any common superscript letters are significantly different at the α = 0.05 level (column proportions compared with the z test; p value adjusted with the Bonferroni method). Respondents could select more than one of the available options under every source type.

**DS source categories**	**Total**	**DS+FO**	**DS-FO**	***p*-value**
**Approval source**				
National Organization for Medicines	9048 (58.0)	641 (64.1)^a^	8407 (57.6)^b^	0.003
Supplier or pharmacist	4310 (27.6)	213 (21.3)^a^	4097 (28.0)^b^	0.003
Supreme Chemical Council	902 (5.8)	61 (6.1)	841 (5.8)	nsd
None of the above/blank	25 (0.2)	1 (0.1)	24 (0.2)	nsd
I don't pay any attention	1532 (9.8)	103 (10.3)	1429 (9.8)	nsd
**Information source**				
Doctor	7289 (46.7)	478 (47.8)	6811 (46.6)	nsd
Pharmacist	6565 (42.1)	491 (49.1)^a^	6074 (41.6)^b^	0.003
Internet	4633 (29.7)	413 (41.3)^a^	4220 (28.9)^b^	0.003
Dietitian	3373 (21.6)	233 (23.3)	3140 (21.5)	nsd
Coach/fitness instructor	2668 (17.1)	184 (18.4)	2484 (17.0)	nsd
Friend	2600 (16.7)	213 (21.3)^a^	2387 (16.3)^b^	0.003
Advertisement	1664 (10.7)	116 (11.6)	1548 (10.6)	nsd
Family	931 (6.0)	54 (5.4)	877 (6.0)	nsd
**Other source**	87 (0.6)	14 (1.4)^a^	73 (0.5)^b^	0.003
Scientific journal/book	46 (0.3)	11 (1.1)^a^	35 (0.2)^b^	0.003
Personal research	20 (0.1)	2 (0.2)	18 (0.1)	nsd
DS company	15 (0.1)	1 (0.1)	14 (0.1)	nsd
Shop	6 (0.1)	0 (0.0)	6 (0.1)	nsd
**Recommendation source**				
Doctor	6611 (42.4)	433 (43.3)	6178 (42.3)	nsd
Pharmacist	4574 (29.3)	357 (35.7)^a^	4217 (28.9)^b^	0.003
Coach	2490 (16.0)	189 (18.9)	2301 (15.8)	nsd
Friend	2453 (15.7)	198 (19.8)^a^	2255 (15.4)^b^	0.003
Dietitian	2282 (14.6)	175 (17.5)	2107 (14.4)	nsd
Internet	2080 (13.3)	210 (21.0)^a^	1870 (12.8)^b^	0.003
Book/magazine/brochure	1084 (6.9)	74 (7.4)	1010 (6.9)	nsd
Family	961 (6.2)	65 (6.5)	896 (6.1)	nsd
None of the above	35 (0.2)	0 (0.0)	35 (0.2)	nsd

Additionally, DS+FO selected a higher number of information (Supplementary Table S3) and recommendation sources (Supplementary Table S4). Also, most of these sources, individually, were selected by more DS+FO (Table 3), even if in certain cases statistical significance was absent (e.g., doctors). In addition, doctors and pharmacists were the top two sources in both DS+FO and DS-FO; yet, hierarchical differences between DS+FO and DS-FO were detected for the 3^rd^ from the top and below sources. For instance, the 3^rd^ top source of information for both groups was the internet (41.3% *vs*. 28.9%). However, as a source of recommendation, the 3^rd^ top source for DS+FO was, again, the internet (21.0%) but for DS-FO it was coaches (15.8%).

### 3.4. Prevalence of use of additional dietary supplements

Every DS type and every individual DS (except for iron) was selected by a significantly higher percentage of DS+FO compared to DS-FO (Table 4), while DS+FO seemed to have used a higher number of DS (Supplementary Table S5). Overall (DS+FO and DS-FO), the DS types selected in descending order were vitamins (77.3%; 82.2% *vs*. 76.9%), metals (54.4%; 64.5% *vs*. 53.7%) and herbs and extracts (50.3%; 68.4% *vs*. 49.1%), followed by “unclassified DS” (49.3%; 72.3% *vs*. 45.8%). Regarding individual DS, for both DS+FO and DS-FO, the 1^st^, 3rd and 4^th^ most frequently chosen DS were multivitamins (54.7% *vs*. 46.1%), vitamin *C* (37.3% *vs*. 29.3%) and green/black tea (32.2% *vs*. 20.6%). However, the 2^nd^ most common DS among DS+FO, was *ω*-3 fatty acids (41.0%), while for DS-FO it was iron (29.8%).

**Table 4.** Absolute and relative frequencies in parenthesis (%) of DS types based on total DS users (Total), dietary supplement (DS) users who had used fish oils (FO) among other DS (DS+FO) and DS users who had used DS but not FO (DS-FO). The *p*-values of chi-square tests of independence between DS types and DS user categories (i.e., DS+FO, DS-FO) after adjustment with Bonferroni correction test, are presented^†^. Within a row, column proportions that do not share any common superscript letters are significantly different at the α = 0.05 level (column proportions compared with the z test; p value adjusted with the Bonferroni method).

**DS type**	**Total**	**DS+FO**	**DS-FO**	***p*-value**
**Vitamins**	12061 (77.3)	823 (82.2)^a^	11238 (76.9)^b^	0.005
Multivitamin	7281 (46.6)	548 (54.7)^a^	6733 (46.1)^b^	0.005
Vitamin *C*	4653 (29.8)	373 (37.3)^a^	4280 (29.3)^b^	0.005
Folic acid	1745 (11.2)	167 (16.7)^a^	1578 (10.8)^b^	0.005
Vitamin *D*	1645 (10.5)	165 (16.5)^a^	1480 (10.1)^b^	0.005
*B* complex vitamin	1518 (9.7)	188 (18.8)^a^	1330 (9.1)^b^	0.005
Vitamin *B12*	1352 (8.7)	171 (17.1)^a^	1181 (8.1)^b^	0.005
Vitamin *E*	1127 (7.2)	127 (12.7)^a^	1000 (6.8)^b^	0.005
Vitamin *A*	954 (6.1)	106 (10.6)^a^	848 (5.8)^b^	0.005
Vitamin *B6*	550 (3.5)	90 (9.0)^a^	460 (3.1)^b^	0.005
Vitamin *K*	488 (3.1)	62 (6.2)^a^	426 (2.9)^b^	0.005
Niacin	237 (1.5)	50 (5.0)^a^	187 (1.3)^b^	0.005
Biotin	236 (1.5)	45 (4.5)^a^	191 (1.3)^b^	0.005
**Metals**	8487 (54.4)	646 (64.5)^a^	7841 (53.7)^b^	0.005
Iron (*Fe*)	4643 (29.7)	283 (28.3)^a^	4360 (29.8)^a^	nsd
Calcium (*Ca*)	2710 (17.4)	256 (25.6)^a^	2454 (16.8)^b^	0.005
Magnesium (*Mg*)	2398 (15.4)	258 (25.8)^a^	2140 (14.7)^b^	0.005
**Mineral complex**	1789 (11.5)	172 (17.2)^a^	1617 (11.1)^b^	0.005
Potassium (*K*)	802 (5.1)	104 (10.4)^a^	698 (4.8)^b^	0.005
Zinc (*Zn*)	588 (3.8)	110 (11.0)^a^	478 (3.3)^b^	0.005
Selenium (*Se*)	314 (2.0)	56 (5.6)^a^	258 (1.8)^b^	0.005
Manganese (*Mn*)	290 (1.9)	43 (4.3)^a^	247 (1.7)^b^	0.005
Sodium (*Na*)	261 (1.7)	47 (4.7)^a^	214 (1.5)^b^	0.005
Chromium (*Cr*)	154 (1.0)	34 (3.4)^a^	120 (0.8)^b^	0.005
Copper (*Cu*)	139 (0.9)	24 (2.4)^a^	115 (0.8)^b^	0.005
Cobalt (*Co*)	118 (0.8)	14 (1.4)^a^	104 (0.7)^a^	nsd
**Herbs or extracts**	7856 (50.3)	685 (68.4)^a^	7171 (49.1)^b^	0.005
Green/black tea	3327 (21.3)	322 (32.2)^a^	3005 (20.6)^b^	0.005
Spirulina	2724 (17.5)	292 (29.2)^a^	2432 (16.6)^b^	0.005
Hippophae	2202 (14.1)	281 (28.1)^a^	1921 (13.2)^b^	0.005
Aloe vera	1790 (11.5)	209 (20.9)^a^	1581 (10.8)^b^	0.005
Herb combination	1753 (11.2)	205 (20.5)^a^	1548 (10.6)^b^	0.005
Berries	1321 (8.5)	161 (16.1)^a^	1160 (7.9)^b^	0.005
Echinacea	1073 (6.9)	113 (11.3)^a^	960 (6.6)^b^	0.005
Ginseng	903 (5.8)	128 (12.8)^a^	775 (5.3)^b^	0.005
Garlic	777 (5.0)	89 (8.9)^a^	688 (4.7)^b^	0.005
Gingko	366 (2.3)	58 (5.8)^a^	308 (2.1)^b^	0.005
Grape extract	243 (1.6)	44 (4.4)^a^	199 (1.4)^b^	0.005
Kava	79 (0.5)	21 (2.1)^a^	58 (0.4)^b^	0.005
**Unclassified DS**	7414 (47.5)	724 (72.3) ^‡ a^	6690 (45.8)^b^	0.005
Protein	3199 (20.5)	293 (29.3)^a^	2906 (19.9)^b^	0.005
Royal jelly	2370 (15.2)	295 (29.5)^a^	2075 (14.2)^b^	0.005
Ω-fatty acid	1775 (11.4)	410 (41.0)^a^	1365 (9.3)^b^	0.005
Creatine	1349 (8.6)	178 (17.8)^a^	1171 (8.0)^b^	0.005
Weight loss or fat-burner	1292 (8.3)	169 (16.9)^a^	1123 (7.7)^b^	0.005
Energy drinks	1246 (8.0)	132 (13.2)^a^	1114 (7.6)^b^	0.005
Amino acid	1163 (7.5)	142 (14.2)^a^	1021 (7.0)^b^	0.005
Fish oil	1001 (6.4)	1001 (100.0)	0 (0.0)	0.005
Carnitine	887 (5.7)	135 (13.5)^a^	752 (5.1)^b^	0.005
Coenzyme *Q10*	714 (4.6)	163 (16.3)^a^	551 (3.8)^b^	0.005
Glucosamine/chondroitin	286 (1.8)	80 (8.0)^a^	206 (1.4)^b^	0.005
Melatonin	151 (1.0)	43 (4.3)^a^	108 (0.7)^b^	0.005
Α-Lipoic acid	151 (1.0)	43 (4.3)^a^	108 (0.7)^b^	0.005
Other DS	171 (1.1)	19 (1.9)^a^	152 (1.0)^a^	nsd

Note: nsd: non-significant difference (*p* > 0.05); ^†^ Respondents could select more than one of the available options. ^‡^ Percentage of DS+FO who used at least one DS from the “unclassified” DS category without taking into account “fish oils”.

### 3.5. Dietary supplement purchasing parameters

The top parameters for buying DS were the popularity of the manufacturing company (48.8%), the certification of the product's effect via studies (38.7%), the provision of information regarding side effects (34.1%) and the product price-content relationship (31.2%, Table 5, overall). Almost every one of the listed parameters was chosen significantly more frequently by DS+FO, who also tended to consider a higher combination of parameters simultaneously (Supplementary Table S6). Also, while for both DS+FO and DS-FO, the two top considerations were the aforementioned popularity (61.6% vs 48.0%, respectively) and certifications (49.4% vs 37.9%, respectively), the 3^rd^ most important parameter for DS+FO was the product/price relationship (41.2%) and for DS-FO, the provision of information regarding side effects (33.9%).

**Table 5.** Absolute and relative frequencies in parenthesis (%) of the parameters of buying dietary supplements (DS) based on total DS users (Total), DS users who had used fish oils (FO) among other DS (DS+FO) and DS users who had used DS but not FO (DS-FO). The *p*-values of chi-square tests of independence between DS use parameters and DS categories (i.e., DS+FO, DS-FO), after adjustment with Bonferroni correction test, are presented. Within a row, column proportions that do not share any common superscript letters are significantly different at the α = 0.05 level (column proportions compared with the z test; p value adjusted with the Bonferroni method).

**Parameters of buying DS**	**Total**	**DS+FO**	**DS-FO**	***p*-value**
Popularity of manufacturing company	7621 (48.8)	616 (61.6)^a^	7005 (48.0)^b^	0.002
Certification of the product's effect via studies	6035 (38.7)	494 (49.4)^a^	5541 (37.9)^b^	0.002
Provision of information regarding side-effects	5318 (34.1)	365 (36.5)	4953 (33.9)	nsd
Product price/content relationship	4866 (31.2)	412 (41.2)^a^	4454 (30.5)^b^	0.002
Form of sold product	1864 (11.9)	162 (16.2)^a^	1702 (11.7)^b^	0.002
Package attractiveness	679 (4.4)	70 (7.0)^a^	609 (4.2)^b^	0.002
**Other parameter/s^†^**	524 (3.4)	30 (3.0)	494 (3.4)	nsd
Opinion of doctor/pharmacist/dietitian	404 (2.6)	17 (1.7)	387 (2.6)	nsd
No parameter	58 (0.4)	2 (0.2)	56 (0.4)	nsd
Information from the internet/friends	51 (0.3)	3 (0.3)	48 (0.3)	nsd
Natural origin	21 (0.1)	2 (0.2)	19 (0.1)	nsd
Notification of National Organization for Medicines	17 (0.1)	2 (0.2)	15 (0.1)	nsd
Nutrient analogy	13 (0.1)	3 (0.3)	10 (0.1)	nsd
Knowledge of fitness instructor	13 (0.1)	2 (0.2)	11 (0.1)	nsd
Country of origin	5 (0.1)	1 (0.1)	4 (0.1)	nsd

Note: nsd: non-significant difference (*p* > 0.05). ^†^Even though “no parameter” was mentioned in the available frame under “Other parameter”, they were not included in the calculation of the relative and absolute frequency displayed in the “Other parameter” row. ^‡^Respondents could select more than one of the available options.

### 3.6. MLR analysis

The first two columns of Table 6 focus on the comparison between DS+FO and DS non-users and display the odds of one being a DS+FO compared to being a DS non-user, while the last two columns focus on the comparison between DS+FO and DS-FO by displaying the odds of one being a DS+FO compared to being a DS-FO. Regarding the first comparison, based on Wald's test, it seems that sex was not a predictor of DS use (*p* = 0.578). However, in the second comparison, i.e., being a DS+FO *vs*. a DS-FO, men had 35.5% higher odds of being a DS+FO compared to women.

Regarding age, compared to those >60, those in the age groups 15–20 and 21–30 years old, had lower odds of being a DS+FO compared to being a DS non-user (-65.3% and -36.7%, respectively) or a DS-FO (-59.7% and -50.4%, respectively). Additionally, those between 31–40 years old had lower odds of being DS+FO but only when compared to being DS-FO (-35.7%).

As for BMI, in comparison with those of a normal BMI, those who were underweight had higher odds of being a DS+FO compared to being a DS non-user (123.2%) or a DS-FO (89.0%).

For income, compared with those with a monthly income of >2000 €, those in the 500–1000 € group had fewer odds of being a DS+FO compared to being a DS non-user (-37.5%) or a DS-FO (-34.0%), while the other income groups could not significantly predict DS+FO usage in either way (*p* > 0.050).

Regarding educational attainment, compared with those with postgraduate education, those with primary and secondary education had lower odds of being a DS+FO compared to being a DS non-user (-56.2% and -35.3%, respectively). However, when comparing the odds of being a DS+FO with being a DS-FO, significantly lower odds were observed only for those with secondary education (-22.8%).

As for employment status, compared with unemployed respondents, private employees had 37.7% higher odds of being a DS+FO compared to being a DS non-user. However, none of the employment categories could significantly predict being a DS+FO when compared to being a DS-FO (*p* > 0.050). With regards to exercise, compared with exercisers, non-exercisers had lower odds of being a DS+FO compared to being a DS non-user or a DS-FO (-51.8% and -28.2%).

Last but not least, regarding the type of diet followed, in comparison with those who follow a “mixed-unrestricted diet”, higher odds of being a DS+FO compared to being a DS non-user were observed (in a descending order) for those who follow “other diets” (i.e., other than the ones listed in the questionnaire) (332.9%), vegan/vegetarian (148.8%), starch/carbohydrate restricted (137.5%), lacto-ovo-vegetarian (72.7%) and fat restricted (35.9%) diets. However, when comparing the odds of being a DS+FO compared to being a DS-FO, only those who followed a starch/carbohydrate restricted diet had higher odds towards being a DS+FO (51.5%).

**Table 6.** Transformed results of the MLR with Wald's test *p*-values, the adjusted odds ratios (OR) and the corresponding confidence intervals (CI) for the relationship of DS+FO *vs*. DS non-users and DS+FO *vs*. DS-FO with the independent variables (reference category: DS+FO). DS+FO: DS users who had used fish oils among other DS, DS-FO: DS users who had used DS but not FO, DS non-users: respondents who had never used DS. ns: non-significant at α = 0.05.

**Variable**	**DS+FO/DS non-user**		**DS+FO/DS-FO**	
	***OR* (95% *CI*)**	***p*-value**	***OR* (95% *CI*)**	***p*-value**
**Sex**				
Men	0.961 (0.835–1.106)	ns	1.355 (1.179–1.558)	0.000
Women	-		-	
**Age (years old)**				
15–20	0.347 (0.224–0.538)	0.000	0.403 (0.260–0.625)	0.000
21–30	0.633 (0.437–0.917)	0.016	0.496 (0.342–0.719)	0.000
31–40	0.899 (0.628–1.287)	ns	0.643 (0.449–0.922)	0.016
41–50	0.871 (0.606–1.252)	ns	0.720 (0.501–1.035)	ns
51–60	1.075 (0.743–1.555)	ns	1.019 (0.704–1.477)	ns
>60	-		-	
**BMI**				
Underweight	2.232 (1.595–3.125)	0.000	1.890 (1.362–2.625)	0.000
Overweight	1.063 (0.912–1.239)	ns	1.019 (0.876–1.186)	ns
Obese	1.045 (0.821–1.330)	ns	0.912 (0.717–1.157)	ns
Normal weight	-		-	
**Monthly income (€)**				
<500	0.674 (0.448–1.012)	ns	0.798 (0.536–1.189)	ns
500–1000	0.625 (0.426–0.915)	0.016	0.660 (0.455–0.958)	0.029
1001–1500	0.692 (0.467–1.024)	ns	0.742 (0.506–1.089)	ns
1501–2000	0.842 (0.525–1.350)	ns	0.936 (0.590–1.486)	ns
>2000	-		-	
**Education level**				
Primary	0.438 (0.283–0.679)	0.000	0.855 (0.551–1.326)	nsd
Secondary	0.647 (0.515–0.813)	0.000	0.772 (0.618–0.965)	0.023
Tertiary	0.845 (0.680–1.049)	ns	0.941 (0.761–1.161)	nsd
Postgraduate	-		-	
**Employment status**				
Student	1.064 (0.817–1.383)	ns	0.835 (0.642–1.087)	ns
Private employee	1.377 (1.065–1.783)	0.015	0.969 (0.750–1.252)	ns
Public employee	1.289 (0.962–1.727)	ns	1.035 (0.774–1.383)	ns
Freelancer	1.274 (0.968–1.675)	ns	0.898 (0.684–1.181)	ns
Farmer	0.718 (0.463–1.112)	ns	0.674 (0.434–1.047)	ns
Unemployed	-		-	
**Exercise**				
Non-exerciser	0.482 (0.416–0.558)	0.000	0.718 (0.621–0.831)	0.000
Exerciser	-		-	
**Type of diet**				
Fat restricted	1.359 (1.134–1.626)	0.001	0.987 (0.827–1.179)	nsd
Starch/carbohydrate restricted	2.375 (1.802–3.125)	0.000	1.515 (1.161–1.976)	0.002
Calorie restricted	1.110 (0.884–1.393)	nsd	0.992 (0.792–1.242)	nsd
Vegan/vegetarian	2.488 (1.629–3.802)	0.000	1.473 (0.980–2.217)	nsd
Lacto-vegetarianism	1.149 (0.496–2.667)	nsd	0.756 (0.331–1.730)	nsd
Lacto-ovo-vegetarianism	1.727 (1.116–2.674)	0.014	1.192 (0.777–1.825)	nsd
Other diet	4.329 (1.370–13.699)	0.013	2.237 (0.769–6.494)	nsd
Mixed unrestricted	-		-	

## 4. Discussion

### 4.1. Prevalence of FO usage

Based on data collected during 2018 to 2019, we found that 6.5% of DS users (or 3.5% of the overall sample) had used or are currently using FO. Similarly, both the European Prospective Investigation into Cancer and Nutrition (EPIC) study (1995–2000 data) [8] and a more recent study by Kanellou et al. (2013–2014 data) [12] on a representative Greek sample found a usage rate of less than 5.0% (according to 24-hour dietary recalls for both and, additionally for the second study, food propensity questionnaires). Similarly, an Australian study found that 6.0% of its participants were using FO [27], while a more recent one found that 9.2% of its respondents had used FO preparations (without added nutrients; 2^nd^ most used DS) [2]. However, the percentages of FO users in other countries (including those in EPIC) have been shown to be considerably higher in certain cases. For instance, regarding “current or regular use”, the percentage of FO users was 21.9% in New Zealand (2015 data) [3], 31.6% in the United Kingdom (2006–2010 data) [31] and 44.7% in Norway (1998 data; women only) [4]. In fact, a UK study (2018 data) found that of those who were current DS users, 35.0% were currently using FO and 58.0% had taken them in the past [6]. Last but not least, Asian studies have also shown high usage rates in both adults and children. Specifically, Parmenter et al. sampled China, Thailand and Vietnam throughout 2019 and found that 23.0% of their adult respondents had used a specific FO product during the last year [32]. Similarly, they found that 35.0% of children (<18 years old) had been given that FO product by their parents [33]. However, an earlier 2010 Chinese study found that 69.0% of the participating parents gave their kindergarten-aged children cod FO in the last three months [5].

### 4.2. Additional DS use, label comprehension and opinions about the adequacy of diet

Our analysis showed that DS users are more likely to think that nutrient intake from a diet without DS is inadequate, while DS non-users think the opposite. Indeed, in our previous study we found that following a proper diet was the 3^rd^ top reason for not using DS (selected by 33.2% of DS non-users), while “nutrient deficiencies” was the 2^nd^ top reason for using DS (selected by 35.9% of DS users) [9]. Furthermore, DS users, compared to DS non-users, were significantly more likely to declare that they can draw certain conclusions about DS just by reading their labels (i.e., about the relative importance of DS for them and detection of approved or prohibited ingredients or DS).

Now, the above results were even more prevalent among DS+FO, compared to DS-FO. In fact, most DS+FO think that the nutrient intake from foods is definitely inadequate (36.6% *vs*. 30.7% of DS-FO and 9.5% of DS non-users). At the same time, every DS (except for iron) was used by a significantly higher percentage of DS+FO, when compared to DS-FO. Moreover, 91.9% of DS+FO have used three or more DS in their lifetime, compared to only 62.7% of DS-FO.

On a related note, a 2014 review by Dickinson and MacKay showed that it was more probable for DS users to have a healthier dietary pattern, when compared to DS non-users [34]. Similarly, certain studies have pointed out differences in dietary patterns between FO users and FO non-users. For example, FO consumption was positively associated with consumption of fish [3],[4],[33], fruits and vegetables [4] and a higher intake of red and processed meat [10]. However, a negative association was noted between FO and nut consumption [3]. Furthermore, history of FO use has been positively associated with use of additional DS in other studies as well [4],[31],[33].

### 4.3. Views about DS

Studies, including our previous work, have showed that DS users were more likely to have positive views towards DS compared to DS non-users [9]. For instance, in a national study in the UK, a general consideration of DS as risk-free was observed [6], while in a Dutch one, DS non-users had a more risk-averse stance [26]. Similarly, we found that such favorable beliefs were reported significantly more often by DS+FO. For example, more DS+FO believed that DS are generally harmless and that they were necessary for all ages. Other studies have revealed a positive relationship between FO usage and belief in their safety, efficacy and its scientific proof etc. [32],[33] or even a generally more positive attitude [35].

### 4.4. DS purchasing parameters and sources of information and recommendation

Overall, the two most commonly considered DS purchasing parameters were the manufacturer's popularity (48.8%) and the existence of certifications of the effects of said DS (38.7%). However, a significantly higher percentage of DS+FO seemed to take into consideration almost every listed parameter. Moreover, ≅65.0% of DS+FO considered two or more parameters, compared to ≅45.0% of DS-FO. Characteristically, a significantly higher percentage of DS+FO cares about the popularity, the certifications and the price/content relationship. In fact, this last parameter was the 3^rd^ most commonly cited parameter among DS+FO, while for DS-FO it was the provision of information regarding side-effects. The above results suggest that DS+FO are more likely to be subjectively knowledgeable and engaged in the world of DS, to be advocates of DS and show trust in them as seen by the top selected considerations during DS shopping, i.e., popularity, proof of efficacy and affordability, compared to safety precautions. Interpretively, however, popularity might represent a seal of approval towards DS, stemming from the healthcare professional or consumer community, ensuring their safety, efficacy and quality. It is underlined that a higher percentage of DS+FO comes in contact with almost every source of DS information/recommendation (e.g., pharmacists and the internet). Also, around 48.0% and 65.0% of DS+FO–*vs*. 33.0% and 55.0% of DS-FO–are receivers of two or more recommendation and information sources respectively. This further reinforces the previous observations.

In a recent New Zealand study, brand, price and quality were reported to affect the decisions of around 20.0%–35.0% of their respondents [3]. A review by Teoh et al. showed that fundamental factors, with regards to the consumption of nutraceuticals, are the belief in their safety and efficacy, health professional's guidance and the family/friend cycle with cost being a barrier [36]. However, a study pointed out that cost was not significantly associated with lower FO consumption [32]. Α UK study showed that consumers enjoy DS without much concern, given that they shop from well reputed retailers and consume DS responsibly. Also, it was revealed that recommendations and online reviews from professionals (healthcare/fitness), DS users or family/friend cycle are of cardinal importance for the decisions of DS interested individuals [6]. Indeed, while healthcare professionals as sources are reported at considerable rates in certain studies, it seems that they closely contend with the influence of non-healthcare sources (e.g., friends, internet, TV, etc.) [37],[38]. The same goes for FO users specifically [3], although studies have shown healthcare professionals to either have a small effect [39] or no significant effect altogether [32],[33], unlike the social [32],[33] or familial cycle [32].

### 4.5. Sources of DS approval

Despite the apparently more active interest of DS+FO, both DS+FO and DS-FO seem to not be aware of the legislative background of DS. Specifically, ≅90.0% of each group reported checking whether DS are approved by at least one of the listed sources, i.e., the National Organization of Medicines (NOM; 58.0%), the supplier or pharmacist (27.6%) and the Supreme Chemical Council (5.8%). The importance of this result lies within the fact that DS are not approved by a government authority in Greece before being marketed. Indeed, DS are under the competent authority of NOM. However, before a DS is released into the market, NOM must receive a notification letter by their manufacturer, which states specific information, e.g., quantitative and qualitative data about said DS. Henceforth, a notification number, instead of approval indicative items (e.g., “approved by NOM”), is assigned to the DS before it enters the market. In fact, such approval indications regarding their safety, efficacy or quality are considered illegal and misleading [40]. Therefore, even if our DS users had in mind the notification number instead of approval with its strict definition, it is questionable whether they are aware of the relative legislative background.

### 4.6. Profile of DS+FO

Previously, we found certain characteristics to be significant determinants of DS use, i.e., being a woman, a middle aged-man, an older woman, having a higher income as a man, an abnormal BMI as a woman, having higher education, exercising, being employed (with a few job exceptions) and following a special diet (e.g., vegan/vegetarian) [9]. In the current study, we found that being a DS+FO instead of a DS-FO was more likely for men. Also, higher likelihood for one being a DS+FO compared to being a DS-FO or a DS non-user was detected for those who were older, underweight, outside of the 500–1000 € monthly income range, with higher education and exercisers, with employment status not playing such a significant role.

Below, findings of similar studies are discussed. However, their interpretation requires caution as most of these studies have compared FO users with FO non-users, regardless of whether these FO non-users were DS-FO or DS non-users [3],[27],[31]–[33], while we made that distinction. Contrary to our results, the majority of studies have found FO usage to be more likely among women [3],[13],[27],[31], while less studies pointed at men [10],[35] or to an insignificant influence of sex [32],[33]. However, our results regarding the influence of age, income, education and exercise seem to agree with the existing literature, except for BMI–for which mixed results were detected–and the type of diet, as it was examined by a different perspective. Specifically, increasing age has been associated with FO use [4],[10],[13],[27],[31],[32],[35], while few studies have found an insignificant association [3],[33]. Regarding income, the majority of studies are roughly aligned with our results, by pointing out to a positive association between income and FO consumption [3],[27],[32],[33]. Similarly, some studies have used the Townsend Index, a material deprivation index, for which lower values indicate higher material possessions and vice versa. Hence, one study has linked high material possession with FO usage [31], while another did not reveal a significant association [10]. However, employment status, in general terms, did not play a significant role in predicting FO usage in our study. Studies do not report homogenous results regarding the influence of BMI. A study has showed FO users to have slightly higher BMI than FO non-users [10]. Meanwhile, studies have either not found a significant association [3] or have linked FO usage with lower [31] or normal BMI [4]. In agreement with our results, most studies reveal that FO usage is positively associated with the education level [4],[13],[32],[33], as one found a more or less negative association [3], while some studies did not reveal a significant influence [10],[27]. Moreover, previous studies have noted a significantly positive association between physical activity and FO use [4],[10], rather than an insignificant one [3]. Finally, regarding the followed type of diet, dietary patterns have been associated with FO usage in the past. For example, studies have shown FO users to be more likely to consume fish [3],[32],[33], while others have shown that FO users follow healthier diets overall [4],[28].

A fundamental limitation of our study is that we defined a respondent as a DS user if they had used a DS at least once during their entire lifetime and not in a specific timeframe (e.g., last week). In contrast, demographic and behavioral information (e.g., attitude) reflected data at the time of questionnaire completion. Therefore, a cross-tabulation between DS use, as we defined it, and demographic and behavioral data could not necessarily produce realistic results for a number of reasons. For example, a currently habitual user of DS differs from a respondent who had used DS only once in his lifetime. However, they would both be considered DS users, while the second one is practically not. This affects the division of DS users between DS+FO and DS-FO in a similar manner, since even the slightest use of FO would label a respondent as a DS+FO, even if they were currently not using them, leading to biased results.

## 5. Conclusions

Several predictors of FO use and behavioral patterns were identified. Compared to DS non-users, more DS users, especially DS+FO, believe that a diet without DS is inadequate, have beliefs favorable towards DS and are subjectively knowledgeable regarding DS label comprehension. Among DS users, almost every DS purchasing parameter and source of information/recommendation was selected by a higher percentage of DS+FO. Meanwhile, DS+FO are more likely to consider a higher number of purchasing parameters and be receivers of more sources of information/recommendations, making them more involved in DS. However, knowledge gaps regarding the legislative background of DS were revealed, regardless of DS use. At the same time, many respondents, overall, seem to seek professional involvement regarding the recommendation of DS.

## Use of AI tools declaration

The authors declare they have not used Artificial Intelligence (AI) tools in the creation of this article.
