# Supplementary material for: Fish oil users of Greece: Predictors, knowledge and habits regarding dietary supplement use
Source: AIMS Public Health. 2023 Nov 13;10(4):896–917. doi: 10.3934/publichealth.2023058 (PMC10764963; doi:10.3934/publichealth.2023058)
Supplement: Supplementary file 1 [file publichealth-10-04-058-s001.pdf]

Public Health

AIMS Public Health, 10(4): 896–917.
DOI: 10.3934/publichealth.2023058
Received: 17 July 2023
Revised: 16 October 2023
Accepted: 29 October 2023
Published: 13 November 2023

http://www.aimspress.com/journal/aimsph

*Research article*

# Fish oil users of Greece: Predictors, knowledge and habits regarding dietary supplement use

**Panagiotis-David Soukiasian[1], Zacharenia Kyrana[2], Konstantina Gerothanasi[1], Efstratios Kiranas[1] and Lambros E. Kokokiris[1,*]**

[1] Department of Nutritional Sciences and Dietetics, School of Health Sciences, International Hellenic University, 57400 Thessaloniki, Greece
[2] Laboratory of Agronomy, Faculty of Agriculture, Forestry and Natural Environment, School of Agriculture, Aristotle University of Thessaloniki, 54124 Thessaloniki, Greece

* **Correspondence:** Email: lamprosk@ihu.gr; Tel: +306977354054.

# Supplementary

**Table S1.** Absolute and relative frequencies in parenthesis (%) of dietary supplement (DS) label comprehension and views about DS based on total respondents (Total), those who had used a DS at least once in their lifetime (DS users), and those who had never used DS (DS non-users). The *p*-values of chi-square tests of independence between given answers and DS use categories (i.e., DS users and DS non-users) after adjustment with the Bonferroni correction test are presented. Within a row, column proportions that do not share any common superscript letters are significantly different at the α = 0.05 level (column proportions compared with the z test; p value adjusted with the Bonferroni method).

| DS label comprehension and views about DS | Total (28102) | **DS users** (15608) | **DS non-users** (12494) | ***p*-value** |
|---|---|---|---|---|
| Are nutrients from foods enough to ensure good health? | | | | 0.000 |
| Yes | 9891 (35.2) | 4337 (27.8)[a] | 5554 (44.5)[b] | |
| Maybe yes | 9835 (35.0) | 5239 (33.6)[a] | 4596 (36.8)[b] | |
| No | 6042 (21.5) | 4854 (31.1)[a] | 1188 (9.5)[b] | |
| I don’t know | 2334 (8.3) | 1178 (7.5)[a] | 1156 (9.3)[b] | |
| Can you understand whether DS are important for you by reading their labels? | | | | 0.000 |
| Yes | 10878 (38.7) | 7246 (46.4)[a] | 3632 (29.1)[b] | |
| Maybe yes | 10857 (38.6) | 5920 (37.9)[a] | 4937 (39.5)[b] | |
| No | 6367 (22.7) | 2442 (15.6)[a] | 3925 (31.4)[b] | |
| Can you recognize which ingredients or DS are approved if you read the DS’s label? | | | | 0.000 |
| Yes | 6614 (23.5) | 4366 (28.0)[a] | 2248 (18.0)[b] | |
| Maybe yes | 9142 (32.5) | 5486 (35.1)[a] | 3656 (29.3)[b] | |
| No | 12346 (43.9) | 5756 (36.9)[a] | 6590 (52.7)[b] | |
| Agreement with the statement | | | | |
| DS are necessary for all ages | 4061 (14.5) | 3012 (19.3)[a] | 1049 (8.4)[b] | 0.000 |
| DS are generally harmless | 7012 (25.0) | 4600 (29.5)[a] | 2412 (19.3)[b] | 0.000 |
| Regular DS use can prevent many ailments | 6083 (21.6) | 4005 (25.7)[a] | 2078 (16.6)[b] | 0.000 |
| DS can prevent cancer | 1276 (4.5) | 792 (5.1)[a] | 484 (3.9)[b] | 0.000 |
| DS must be recommended by doctors just like medicines are | 17007 (60.5) | 8121 (52.0)[a] | 8886 (71.1)[b] | 0.000 |
| None of the above | 275 (1.0) | 88 (0.6)[a] | 187 (1.5)[b] | 0.000 |

**Table S2.** Absolute frequency (AF), relative frequency (RF, %) and cumulative relative frequency (CRF, %) of number of approval sources selected by total dietary supplement (DS) users (Total), DS users who had used fish oils (FO) among other DS (DS+FO) and DS users who had used DS but not FO (DS-FO).

| Nb of selected approval sources | Total (*n* = 15.608) | | | **DS+FO** (*n* = 1.001) | | | **DS-FO** (*n* = 14.607) | | |
|---|---|---|---|---|---|---|---|---|---|
| | AF | RF (%) | CRF (%) | AF | RF (%) | CRF (%) | AF | RF (%) | CRF (%) |
| 3 | 15 | 0.1 | 0.1 | 3 | 0.3 | 0.3 | 12 | 0.1 | 0.1 |
| 2 | 173 | 1.1 | 1.2 | 11 | 1.1 | 1.4 | 162 | 1.1 | 1.2 |
| 1 | 13869 | 88.9 | 90.1 | 884 | 88.3 | 89.7 | 12985 | 88.9 | 90.1 |
| 0 | 1551 | 9.9 | 100.0 | 103 | 10.3 | 100.0 | 1448 | 9.9 | 100.0 |
| Total | 15608 | 100.0 | | 1001 | 100.0 | | 14607 | 100.0 | |

**Table S3.** Absolute frequency (AF), relative frequency (RF, %) and cumulative relative frequency (CRF, %) of number (nb) of information sources selected by total dietary supplement (DS) users (Total), DS users who had used fish oils (FO) among other DS (DS+FO) and DS users who had used DS but not FO (DS-FO).

| Nb of selected information sources | Total (*n* = 15.608) | | | **DS+FO** (*n* = 1.001) | | | **DS-FO** (*n* = 14.607) | | |
|---|---|---|---|---|---|---|---|---|---|
| | AF | RF (%) | CRF (%) | AF | RF (%) | CRF (%) | AF | RF (%) | CRF (%) |
| 8 | 14 | 0.1 | 0.1 | 1 | 0.1 | 0.1 | 13 | 0.1 | 0.1 |
| 7 | 22 | 0.1 | 0.2 | 4 | 0.4 | 0.5 | 18 | 0.1 | 0.2 |
| 6 | 71 | 0.5 | 0.7 | 12 | 1.2 | 1.7 | 59 | 0.4 | 0.6 |
| 5 | 267 | 1.7 | 2.4 | 32 | 3.2 | 4.9 | 235 | 1.6 | 2.2 |
| 4 | 857 | 5.5 | 7.9 | 91 | 9.1 | 14.0 | 766 | 5.2 | 7.5 |
| 3 | 2568 | 16.5 | 24.3 | 187 | 18.7 | 32.7 | 2381 | 16.3 | 23.8 |
| 2 | 4842 | 31.0 | 55.4 | 329 | 32.9 | 65.5 | 4513 | 30.9 | 54.7 |
| 1 | 6967 | 44.6 | 100.0 | 345 | 34.5 | 100.0 | 6622 | 45.3 | 100.0 |
| Total | 15608 | 100.0 | | 1001 | 100.0 | | 14607 | 100.0 | |

**Table S4.** Absolute frequency (AF), relative frequency (RF, %) and cumulative relative frequency (CRF, %) of number (nb) of recommendation sources selected by total dietary supplement (DS) users (Total), DS users who had used fish oils (FO) among other DS (DS+FO) and DS users who had used DS but not FO (DS-FO).

| Nb of selected recommendation sources | Total (*n* = 15.608) | | | **DS+FO** (*n* = 1.001) | | | **DS-FO** (*n* = 14.607) | | |
|---|---|---|---|---|---|---|---|---|---|
| | AF | RF (%) | CRF (%) | AF | RF (%) | CRF (%) | AF | RF (%) | CRF (%) |
| 8 | 1 | 0.0 | 0.0 | 0 | 0.0 | 0.0 | 1 | 0.0 | 0.0 |
| 7 | 1 | 0.0 | 0.0 | 0 | 0.0 | 0.0 | 1 | 0.0 | 0.0 |
| 6 | 13 | 0.1 | 0.1 | 4 | 0.4 | 0.4 | 9 | 0.1 | 0.1 |
| 5 | 34 | 0.2 | 0.3 | 6 | 0.6 | 1.0 | 28 | 0.2 | 0.3 |
| 4 | 219 | 1.4 | 1.7 | 32 | 3.2 | 4.2 | 187 | 1.3 | 1.5 |
| 3 | 1068 | 6.8 | 8.6 | 124 | 12.4 | 16.6 | 944 | 6.5 | 8.0 |
| 2 | 3955 | 25.3 | 33.9 | 312 | 31.2 | 47.8 | 3643 | 24.9 | 32.9 |
| 1 | 10282 | 65.9 | 99.8 | 523 | 52.2 | 100.0 | 9759 | 66.8 | 99.8 |
| 0 | 35 | 0.2 | 100.0 | 0 | 0.0 | 100.0 | 35 | 0.2 | 100.0 |
| Total | 15608 | 100.0 | | 1001 | 100.0 | | 14607 | 100.0 | |

**Table S5.** Absolute frequency (AF), relative frequency (RF, %) and cumulative relative frequency (CRF, %) of number of dietary supplements (DS) selected by total DS users (Total), DS users who had used fish oils (FO) among other DS (DS+FO) and DS users who had used DS but not FO (DS-FO).

| Number of selected DS | Total (*n* = 15.608) | | | **DS+FO** (*n* = 1.001) | | | **DS-FO** (*n* = 14.607) | | |
|---|---|---|---|---|---|---|---|---|---|
| | AF | RF (%) | CRF (%) | AF | RF (%) | CRF (%) | AF | RF (%) | CRF (%) |
| 50 | 0 | 0.0 | 0.0 | 0 | 0.0 | 0.0 | 0 | 0.0 | 0.0 |
| 49 | 2 | 0.0 | 0.0 | 2 | 0.2 | 0.2 | 0 | 0.0 | 0.0 |
| 48 | 0 | 0.0 | 0.0 | 0 | 0.0 | 0.2 | 0 | 0.0 | 0.0 |
| 47 | 0 | 0.0 | 0.0 | 0 | 0.0 | 0.2 | 0 | 0.0 | 0.0 |
| 46 | 1 | 0.0 | 0.0 | 1 | 0.1 | 0.3 | 0 | 0.0 | 0.0 |

*Continued on next page*

| Number of selected DS | Total (*n* = 15.608) | | | **DS+FO** (*n* = 1.001) | | | **DS-FO** (*n* = 14.607) | | |
|---|---|---|---|---|---|---|---|---|---|
| | AF | RF (%) | CRF (%) | AF | RF (%) | CRF (%) | AF | RF (%) | CRF (%) |
| 45 | 0 | 0.0 | 0.0 | 0 | 0.0 | 0.3 | 0 | 0.0 | 0.0 |
| 44 | 0 | 0.0 | 0.0 | 0 | 0.0 | 0.3 | 0 | 0.0 | 0.0 |
| 43 | 0 | 0.0 | 0.0 | 0 | 0.0 | 0.3 | 0 | 0.0 | 0.0 |
| 42 | 0 | 0.0 | 0.0 | 0 | 0.0 | 0.3 | 0 | 0.0 | 0.0 |
| 41 | 0 | 0.0 | 0.0 | 0 | 0.0 | 0.3 | 0 | 0.0 | 0.0 |
| 40 | 0 | 0.0 | 0.0 | 0 | 0.0 | 0.3 | 0 | 0.0 | 0.0 |
| 39 | 0 | 0.0 | 0.0 | 0 | 0.0 | 0.3 | 0 | 0.0 | 0.0 |
| 38 | 2 | 0.0 | 0.0 | 2 | 0.2 | 0.5 | 0 | 0.0 | 0.0 |
| 37 | 0 | 0.0 | 0.0 | 0 | 0.0 | 0.5 | 0 | 0.0 | 0.0 |
| 36 | 1 | 0.0 | 0.0 | 1 | 0.1 | 0.6 | 0 | 0.0 | 0.0 |
| 35 | 4 | 0.0 | 0.1 | 3 | 0.3 | 0.9 | 1 | 0.0 | 0.0 |
| 34 | 1 | 0.0 | 0.1 | 0 | 0.0 | 0.9 | 1 | 0.0 | 0.0 |
| 33 | 5 | 0.0 | 0.1 | 4 | 0.4 | 1.3 | 1 | 0.0 | 0.0 |
| 32 | 2 | 0.0 | 0.1 | 0 | 0.0 | 1.3 | 2 | 0.0 | 0.0 |
| 31 | 2 | 0.0 | 0.1 | 2 | 0.2 | 1.5 | 0 | 0.0 | 0.0 |
| 30 | 2 | 0.0 | 0.1 | 1 | 0.1 | 1.6 | 1 | 0.0 | 0.0 |
| 29 | 3 | 0.0 | 0.2 | 2 | 0.2 | 1.8 | 1 | 0.0 | 0.0 |
| 28 | 7 | 0.0 | 0.2 | 4 | 0.4 | 2.2 | 3 | 0.0 | 0.1 |
| 27 | 3 | 0.0 | 0.2 | 1 | 0.1 | 2.3 | 2 | 0.0 | 0.1 |
| 26 | 6 | 0.0 | 0.3 | 3 | 0.3 | 2.6 | 3 | 0.0 | 0.1 |
| 25 | 12 | 0.1 | 0.3 | 6 | 0.6 | 3.2 | 6 | 0.0 | 0.1 |
| 24 | 12 | 0.1 | 0.4 | 4 | 0.4 | 3.6 | 8 | 0.1 | 0.2 |
| 23 | 11 | 0.1 | 0.5 | 4 | 0.4 | 4.0 | 7 | 0.0 | 0.2 |
| 22 | 18 | 0.1 | 0.6 | 8 | 0.8 | 4.8 | 10 | 0.1 | 0.3 |
| 21 | 28 | 0.2 | 0.8 | 12 | 1.2 | 6.0 | 16 | 0.1 | 0.4 |
| 20 | 23 | 0.1 | 0.9 | 10 | 1.0 | 7.0 | 13 | 0.1 | 0.5 |
| 19 | 31 | 0.2 | 1.1 | 9 | 0.9 | 7.9 | 22 | 0.2 | 0.7 |
| 18 | 29 | 0.2 | 1.3 | 9 | 0.9 | 8.8 | 20 | 0.1 | 0.8 |
| 17 | 45 | 0.3 | 1.6 | 15 | 1.5 | 10.3 | 30 | 0.2 | 1.0 |
| 16 | 49 | 0.3 | 1.9 | 12 | 1.2 | 11.5 | 37 | 0.3 | 1.3 |
| 15 | 76 | 0.5 | 2.4 | 18 | 1.8 | 13.3 | 58 | 0.4 | 1.7 |
| 14 | 84 | 0.5 | 2.9 | 26 | 2.6 | 15.9 | 58 | 0.4 | 2.1 |
| 13 | 138 | 0.9 | 3.8 | 28 | 2.8 | 18.7 | 110 | 0.8 | 2.8 |
| 12 | 171 | 1.1 | 4.9 | 35 | 3.5 | 22.2 | 136 | 0.9 | 3.7 |
| 11 | 230 | 1.5 | 6.4 | 48 | 4.8 | 27.0 | 182 | 1.2 | 5.0 |
| 10 | 287 | 1.8 | 8.2 | 48 | 4.8 | 31.8 | 239 | 1.6 | 6.6 |
| 9 | 370 | 2.4 | 10.6 | 59 | 5.9 | 37.7 | 311 | 2.1 | 8.7 |
| 8 | 496 | 3.2 | 13.8 | 50 | 5.0 | 42.7 | 446 | 3.1 | 11.8 |
| 7 | 773 | 5.0 | 18.7 | 84 | 8.4 | 51.0 | 689 | 4.7 | 16.5 |
| 6 | 963 | 6.2 | 24.9 | 94 | 9.4 | 60.4 | 869 | 5.9 | 22.5 |
| 5 | 1412 | 9.0 | 34.0 | 101 | 10.1 | 70.5 | 1311 | 9.0 | 31.4 |
| 4 | 2029 | 13.0 | 47.0 | 119 | 11.9 | 82.4 | 1910 | 13.1 | 44.5 |
| 3 | 2753 | 17.6 | 64.6 | 95 | 9.5 | 91.9 | 2658 | 18.2 | 62.7 |
| 2 | 3048 | 19.5 | 84.1 | 57 | 5.7 | 97.6 | 2991 | 20.5 | 83.2 |
| 1 | 2479 | 15.9 | 100.0 | 24 | 2.4 | 100.0 | 2455 | 16.8 | 100.0 |
| Total | 15608 | 100.0 | | 1001 | 100.0 | | 14607 | 100.0 | |

Note: *A very small percentage of DS users (1.1%) used DS that were not included in the provided list ("Other DS"). The filling of the provided space of "other DS" with at least one DS, was counted as one selection.

**Table S6.** Absolute frequency (AF), relative frequency (RF, %) and cumulative relative frequency (CRF, %) of number of selected dietary supplement (DS) purchasing parameters selected by total DS users (Total), DS users who had used fish oils (FO) among other DS (DS+FO) and DS users who had used DS but not FO (DS-FO).

| Nb of selected DS purchasing parameters | Total (*n* = 15.608) | | | **DS+FO** (*n* = 1.001) | | | **DS-FO** (*n* = 14.607) | | |
|---|---|---|---|---|---|---|---|---|---|
| | AF | RF (%) | CRF (%) | AF | RF (%) | CRF (%) | AF | RF (%) | CRF (%) |
| 6 | 59 | 0.4 | 0.4 | 11 | 1.1 | 1.1 | 48 | 0.3 | 0.3 |
| 5 | 130 | 0.8 | 1.2 | 13 | 1.3 | 2.4 | 117 | 0.8 | 1.1 |
| 4 | 599 | 3.8 | 5.0 | 109 | 10.9 | 13.3 | 490 | 3.4 | 4.5 |
| 3 | 2080 | 13.3 | 18.4 | 201 | 20.1 | 33.4 | 1879 | 12.9 | 17.3 |
| 2 | 4585 | 29.4 | 47.8 | 314 | 31.4 | 64.7 | 4271 | 29.2 | 46.6 |
| 1 | 8097 | 51.9 | 99.6 | 351 | 35.1 | 99.8 | 7746 | 53.0 | 99.6 |
| 0 | 58 | 0.4 | 100.0 | 2 | 0.2 | 100.0 | 56 | 0.4 | 100.0 |
| Total | 15608 | 100.0 | | 1001 | 100.0 | | 14607 | 100.0 | |

**Table S7.** Initial results of the MLR with Wald's test *p*-values, the adjusted odds ratios (OR) and the corresponding confidence intervals (CI) for the relationship of DS+FO *vs* DS non-users and DS+FO *vs* DS-FO with the independent variables (reference category: DS+FO). DS+FO: dietary supplement (DS) users who had used fish oils (FO) among other DS, DS-FO: DS users who had used DS but not FO, DS non-users: respondents who had never used DS. ns: non significant at α=0.05.

| Variable | **DS non-user/DS+FO** | | **DS-FO/DS+FO** | |
|---|---|---|---|---|
| | *OR* (95.0% *CI*) | *p*-value | *OR* (95.0% *CI*) | *p*-value |
| **Gender** | | | | |
| Male | 1.041 (0.904–1.197) | ns | 0.738 (0.642–0.848) | 0.000 |
| Female | - | | - | |
| **Age (years old)** | | | | |
| 15–20 | 2.878 (1.858–4.459) | 0.000 | 2.481 (1.600–3.847) | 0.000 |
| 21–30 | 1.579 (1.090–2.287) | 0.016 | 2.015 (1.390–2.920) | 0.000 |
| 31–40 | 1.112 (0.777–1.593) | ns | 1.555 (1.085–2.228) | 0.016 |
| 41–50 | 1.148 (0.799–1.650) | ns | 1.389 (0.966–1.998) | ns |
| 51–60 | 0.930 (0.643–1.346) | ns | 0.981 (0.677–1.421) | ns |
| >60 | - | | - | |
| **BMI** | | | | |
| Underweight | 0.448 (0.320–0.627) | 0.000 | 0.529 (0.381–0.734) | 0.000 |
| Overweight | 0.941 (0.807–1.096) | ns | 0.981 (0.843–1.141) | ns |
| Obese | 0.957 (0.752–1.218) | ns | 1.097 (0.864–1.394) | ns |
| Normal weight | - | | - | |
| **Monthly income** | | | | |
| <500 € | 1.484 (0.988–2.230) | ns | 1.253 (0.841–1.867) | ns |
| 500–1000 € | 1.601 (1.093–2.346) | 0.016 | 1.515 (1.044–2.200) | 0.029 |
| 1001–1500 € | 1.446 (0.977–2.142) | ns | 1.347 (0.918–1.978) | ns |
| 1501–2000 € | 1.188 (0.741–1.906) | ns | 1.068 (0.673–1.696) | ns |
| >2000 € | - | | - | |

*Continued on next page*

| Variable | DS non-user/DS+FO | | DS-FO/DS+FO | |
|---|---|---|---|---|
| | *OR* (95.0% *CI*) | *p*-value | *OR* (95.0% *CI*) | *p*-value |
| **Education level** | | | | |
| Primary education | 2.282 (1.473–3.537) | 0.000 | 1.170 (0.754–1.816) | ns |
| Secondary education | 1.545 (1.230–1.940) | 0.000 | 1.295 (1.036–1.619) | 0.023 |
| Tertiary education | 1.184 (0.953–1.470) | ns | 1.063 (0.861–1.314) | ns |
| Postgraduate education | - | | - | |
| **Employment status** | | | | |
| Student | 0.940 (0.723–1.224) | ns | 1.197 (0.920–1.557) | ns |
| Private employee | 0.726 (0.561–0.939) | 0.015 | 1.032 (0.799–1.334) | ns |
| Public employee | 0.776 (0.579–1.039) | ns | 0.966 (0.723–1.292) | ns |
| Freelancer | 0.785 (0.597–1.033) | ns | 1.113 (0.847–1.461) | ns |
| Farmer | 1.393 (0.899–2.160) | ns | 1.484 (0.955–2.304) | ns |
| Unemployed | - | | - | |
| **Exercise** | | | | |
| Non-exercisers | 2.074 (1.791–2.402) | 0.000 | 1.392 (1.203–1.611) | 0.000 |
| Exercisers | - | | - | |
| **Type of diet** | | | | |
| Fat restricted | 0.736 (0.615–0.882) | 0.001 | 1.013 (0.848–1.209) | ns |
| Starch/carbohydrate restricted | 0.421 (0.320–0.555) | 0.000 | 0.660 (0.506–0.861) | 0.002 |
| Calorie restricted | 0.901 (0.718–1.131) | ns | 1.008 (0.805–1.262) | ns |
| Vegan/vegetarian | 0.402 (0.263–0.614) | 0.000 | 0.679 (0.451–1.020) | ns |
| Lacto-vegetarianism | 0.870 (0.375–2.017) | ns | 1.322 (0.578–3.025) | ns |
| Lacto-ovo-vegetarianism | 0.579 (0.374–0.896) | 0.014 | 0.839 (0.548–1.287) | ns |
| Other diet | 0.231 (0.073–0.730) | 0.013 | 0.447 (0.154–1.300) | ns |
| Mixed unrestricted | - | | - | |

AIMS Press

© 2023 the Author(s), licensee AIMS Press. This is an open access article distributed under the terms of the Creative Commons Attribution License (http://creativecommons.org/licenses/by/4.0)
